# Interatrial block and atrial remodeling assessed using speckle tracking echocardiography

**DOI:** 10.1186/s12872-018-0776-6

**Published:** 2018-02-21

**Authors:** Juan Lacalzada-Almeida, María Manuela Izquierdo-Gómez, Carima Belleyo-Belkasem, Patricia Barrio-Martínez, Javier García-Niebla, Roberto Elosua, Alejandro Jiménez-Sosa, Luis Alberto Escobar-Robledo, Antonio Bayés de Luna

**Affiliations:** 1 Department of Cardiology, Hospital Universitario de Canarias, Ofra s/n, La Cuesta, 38320 La Laguna, Tenerife, Spain; 2 Servicios Sanitarios del Área de Salud de El Hierro, Valle del Golfo Health Center, El Hierro, Spain; 3 Cardiovascular Epidemiology and Genetics Research Group, IMIM (Hospital del Mar Medical Research Institute), Barcelona, Catalonia Spain; 4 CIBER Cardiovascular Diseases (CIBERCV), Barcelona, Spain; 5 Research Unit, Hospital Universitario de Canarias, La Laguna, Santa Cruz de Tenerife, Spain; 6 Fundación Investigación Cardiovascular, Institut Català Ciències Cardiovasculars, Barcelona, Catalonia Spain

**Keywords:** Interatrial block, Speckle tracking echocardiography, Strain, Strain rate, Left atrium

## Abstract

**Background:**

To evaluate the possibility of left atrial (LA) remodeling using speckle tracking echocardiography (STE) in patients with interatrial block (IAB).

**Methods:**

We performed a cross-sectional study with three groups of patients: 56 without IAB, 21 with partial IAB (pIAB), and 22 with advanced IAB (aIAB). Transthoracic echocardiographic (TTE) STE was performed and clinical and echocardiographic findings were analyzed.

**Results:**

TTE showed higher LA volume/body surface area in the patients with IAB. With STE, the absolute value of strain rate during atrial booster pump function (SRa) and early reservoir period (SRs) decreased in the pIAB group and even more in the aIAB group, compared to the group without IAB. The independent variables were the echocardiographic measures of LA size and function. After adjusting for confounders, both multiple linear regression and multivariate multinomial regression showed good correlation with dependent variables: longer P-wave duration on electrocardiography and with the type of IAB, respectively. SRa (*p* < 0.001), SRs (p < 0.001), and maximal peak LA longitudinal strain in the reservoir period (*p* = 0.009) were independently associated with P-wave duration. SRa was also associated with the presence of pIAB (OR = 11.5; 95% confidence interval (CI): 2.7–49.0; *p* = 0.001) and aIAB, (OR = 98.2; 95% CI: 16–120.4; p < 0.001) and SRs was associated with pIAB (OR: 0.03; CI: 0.003–0.29; *p* = 0.003) and with aIAB (OR: 0.008; CI: 0.001–0.12; *p* = 0.004).

**Conclusions:**

IAB correlates directly with structural remodeling and a decrease in the absolute value of LA SRa and SRs determined using STE.

**Electronic supplementary material:**

The online version of this article (10.1186/s12872-018-0776-6) contains supplementary material, which is available to authorized users.

## Background

**Fig. 1:**
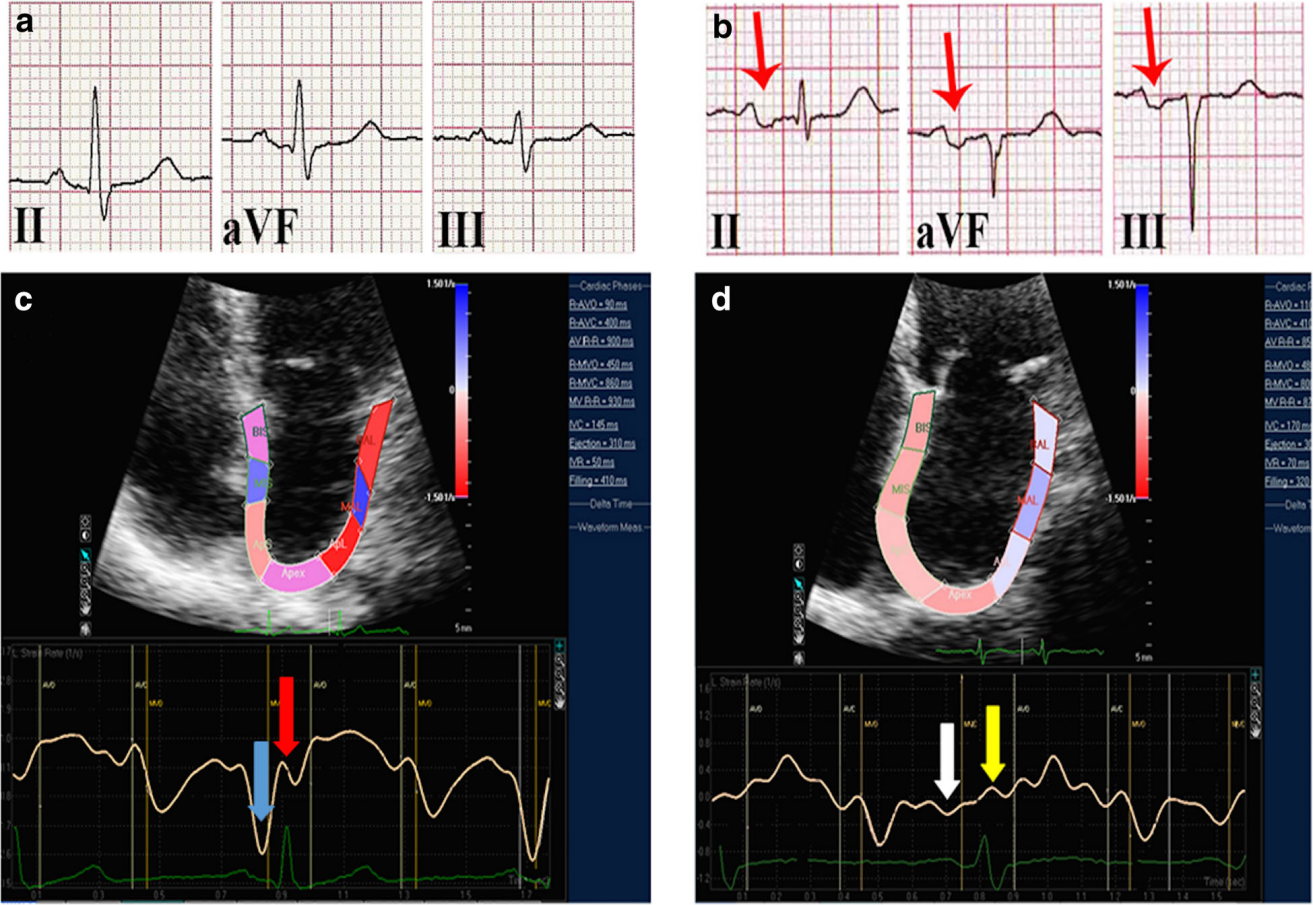
Comparison of ECG and speckle tracking echocardiography of a normal subject and another with advanced interatrial block. ECG (**a**) and left atrial (LA) strain rate (SR) echocardiography (**c**) in normal subjects, with normal SR of LA contraction (blue arrow) and reservoir (red arrow) phases. ECG with advanced interatrial block (**b**); red arrows show biphasic or +/− P wave morphology and with decreased SR of LA contraction (white arrow) and reservoir (yellow arrow) phases (**d**)

Interatrial block (IAB) is an electrical abnormality due to delayed conduction in the Bachmann’s bundle region between the two atria. There are two types of IAB, partial (pIAB) and advanced (aIAB) [1]. In the pIAB, the stimulus is delayed but can cross the septum via the Bachmann’s bundle [2, 3]. Advanced IAB is produced when electrical stimulus conduction is totally blocked in that region; the activation of the left atrium (LA) is then from the right atrium in the caudo-cranial retrograde direction. The pIAB is characterized by a P-wave duration ≥120 milliseconds (ms) on a 12-lead electrocardiogram (ECG) (Fig. 1a) [2, 3]. The diagnosis of aIAB is made by a P-wave duration ≥120 ms and a biphasic or +/− morphology of the P-wave in leads II, III, and aVF of the ECG (Fig. 1b) [2, 3]. Interatrial block is a marker of electromechanical dysfunction of the LA [4] and a substrate for the development of supraventricular arrhythmias in different scenarios [5, 6], mainly atrial fibrillation (AF), which occurs in a condition referred to as Bayés’ Syndrome [7]. Currently, a multicenter registry is tracking these cases to demonstrate this assumption [8].

Several authors have studied alterations in LA wall deformity using speckle tracking echocardiography (STE). Patients undergoing STE before ablation of paroxysmal AF (PAF) have shown reduced LA function [9]. Additionally, decreased peak longitudinal strain rate (SR) during the early reservoir period (SRs) of the LA is related to AF recurrence after electrical cardioversion [10]. In addition, decreased peak longitudinal SR during the period of atrial booster pump function (SRa) of the LA is a predictor of new-onset AF [11].

Changes in atrial function and remodeling and their predisposition to present with new-onset AF [12] have been observed using transthoracic echocardiography (TTE) in patients with IAB [2]. However, to date, no studies have described possible alterations in the LA wall using STE. Our hypothesis was that LA remodeling and altered period function could be observed in patients with IAB in sinus rhythm without a history of supraventricular arrhythmias. Our objective was to determine whether there is an association between IAB and atrial remodeling, with possible alterations in the deformation and functionality of the LA wall, determined using STE.

## Methods

### Study design

We performed a single-cross-sectional study, with prospective and consecutive recruitment of three groups of patients: one group with pIAB, another with aIAB, according to the ECG definition indicated above [2, 3], and a third group without IAB in sinus rhythm (patients with no IAB). Patients with a history of supraventricular arrhythmia verified from their hospital admission, emergency records, or long duration ECG-Holter monitoring were excluded. The study included outpatients aged > 60 years undergoing ECG at our center prospectively between January and May 2016, during the preoperative general surgery assessment. At the same visit, all patients underwent two-dimensional (2D)-Doppler TTE with STE. This study was reviewed and approved by the Clinical Research Ethics Committee of the University Hospital of the Canary Islands (Canary Islands, Tenerife, Spain). Written informed consent was obtained from all patients.

### Electrocardiogram

A 12-lead ECG was performed according to established standards [13]. One experienced operator blinded to the TTE and STE data analyzed the ECGs. The P-wave was measured in the frontal plane leads recorded at the same time, with a digitized ECG using GeoGebra 4.2 software. The ECG image was amplified up to 20 times its original size to define the interval between the earliest and the latest detection of atrial depolarization, defined as a positive or negative deflection, respectively, that deviates from the baseline before the QRS complex. The software allowed us to manually draw lines on the ECG and measure the distance between two points, which was then converted to ms. Participants were categorized into three groups of interest, according to the previously given ECG description.

### Echocardiographic studies

Echocardiograms were digitally stored and analyzed by two experienced operators blinded to the clinical ECG data, using a commercial system (iE33 xMATRIX, Koninklijke Philips NV, Eindhoven, The Netherlands) with a 2–4 MHz multifrequency transducer. Three consecutive beats were recorded during apnea in a cine-loop format. The analysis was performed using an echocardiographic analysis system (Xcelera R2, Philips). Left atrial and left ventricular (LV) measurements, and LV ejection fraction and Doppler variables to quantify LV diastolic function were measured according to standard echocardiographic methods [14, 15]. The LA area was traced in both the apical four- and two-chamber views in the “frame” prior to the mitral valve opening (MVO), using the bi-plane disk summation algorithm to calculate its volume, similar to that used to measure LV volume, adjusted for body surface area (BSA) [14]. The percentage of atrial systolic contribution to total mitral flow (ASCTMF) was determined as a percentage of the integral wave A velocity-time, with respect to the total area of the diastolic velocity-time curve [16].

For the STE of the LA, standard 2D images from apical 4- and 2-chambers views were acquired, with a narrow sector angle (30°–60°) and a frame-rate of 60–90 frames per second [17]. The LA endocardial border was manually traced in both 4- and 2-chamber views using the point-and-click technique, marking two points at both ends of the mitral annulus and a third at the ceiling of the LA, in end systole. The surface epicardial tracing is automatically generated by the system and can be adjusted manually by the operator in cases of tracking failure. Any segments that subsequently failed to track were excluded. The LA myocardium was divided into 12 segments of interest for analysis [17] and longitudinal strain and SR were measured automatically “offline,” using the QLAB Advanced Tissue Motion Quantification (Philips) Release 8.1, equipped with STE analysis software. Global longitudinal strain and SR were the averages of the 12 values obtained for each LA segment. Since the primary variable of interest was the contractile function of the LA, the beginning of the cycle was arbitrarily chosen as the beginning of the P-wave of the ECG [18, 19].

**Fig. 2:**
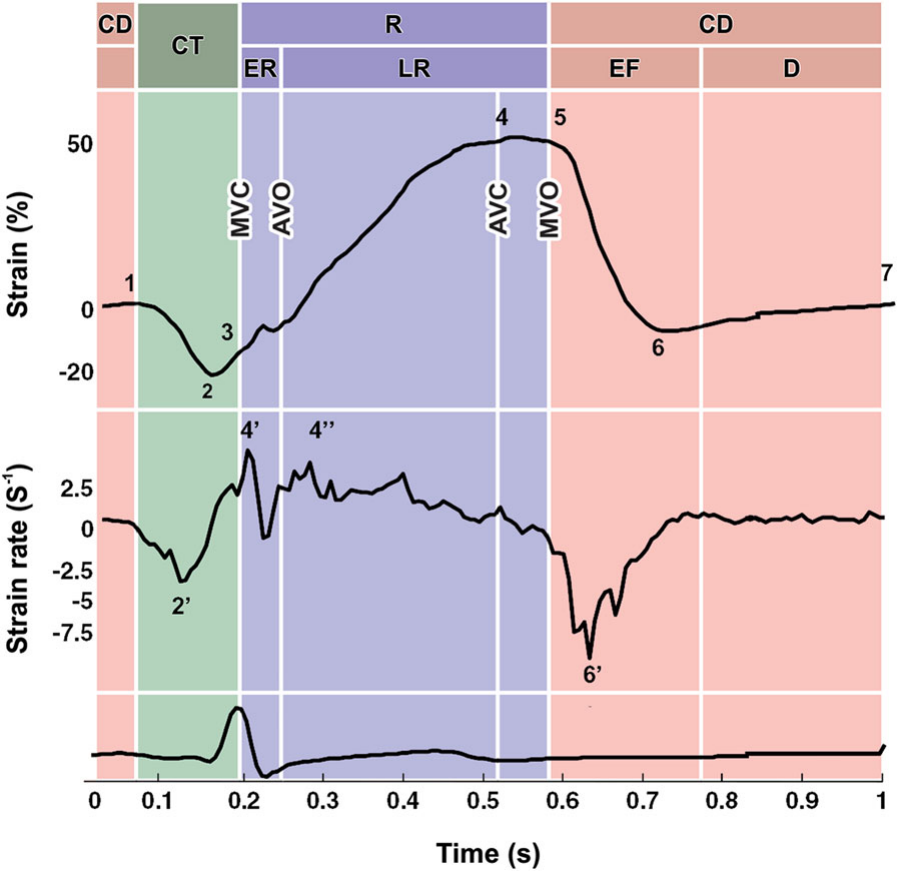
Normal strain and strain rate echocardiography curves in normal subjects. Atrial periods: CD = conduit period; CT = contraction period; R = reservoir period

The periods of the cardiac cycle were determined after obtaining the LA longitudinal strain/SR (Fig. 2), aligning them with the pulsed Doppler spectrum of the LV inflow and outflow tracts, starting at the ECG P-wave. The atrial contraction period (CT) was defined as A-wave duration**,** the LA reservoir period (R) as the interval between mitral valve closure (MVC) and MVO, and the LA conduit period (CD) as the interval between MVO and the onset of the A-wave. Maximum values of longitudinal strain/SR were measured during the LA CT, R, and CD periods [18–20]. In our study, the following parameters of strain and SR were determined (Fig. 2): (1) peak strain point 1, peak strain point 2, and peak SR point 2′ (SRa), during the CT period, (2) in the LA R period the peak strain point 4 or maximal peak LA longitudinal strain (εs LAmax), and peak SR point 4′ (SRs) on early reservoir period (ER), (3) finally, in the CD period, peak strain point 6 and peak SR point 6′ during LV early filling (EF), and peak strain point 7 during diastasis (D).

### Other covariates

Sociodemographic variables and the presence of comorbidities and pharmacological treatments were recorded.

### Statistical analysis

Continuous variables were reported as means and standard deviations or as medians and interquartile ranges according to the nature of the variables (determined using the Shapiro-Wilk test). Categorical variables were reported as frequencies and percentages. Comparisons of continuous variables between groups were carried out using the one-way ANOVA test. Variables that were not normally distributed were compared using the Kruskal-Wallis test. Comparisons between groups for categorical variables were carried out with the chi-square test. Correlations were obtained using the Spearman rank test. Multivariate linear regression analysis adjusted for potential confounders was performed, the dependent variable being P-wave duration and the independent variables being various measures of LA function. To compare the magnitude of these associations, the standardized regression coefficient (b) was calculated. We also used the multinomial logistic regression model, with the dependent variable taking three values ​​ (“pIAB,” “aIAB,” and “patients with no IAB”). The independent variables were the echocardiographic measures of LA size and function. Both the linear and logistic regression models were adjusted for potential confounders, including those variables that proved significant on univariate analysis: age, gender, arterial hypertension, diabetes mellitus, maximal LA volume indexed to body surface area (LAVImax) (mL/m^2^), and ASCTMF. Odds ratio (OR) and 95% confidence intervals (CIs) were calculated as measures of the clinical impact of the predictor variables. Overall differences between models were tested by calculating overall differences in log-likelihood chi-square analysis between models. The ability of the multivariate logistic model to correlate with the presence or absence of IAB was verified using receiver operating characteristic (ROC) curves. The overall accuracy, sensitivity, specificity, and positive and negative predictive values for the optimal cut-off were calculated using the Youden index. Intra- and inter-observer variability was assessed using Bland-Altman analysis. A two-tailed *P* value of < 0.05 was considered to indicate statistical significance. All statistical analyses were performed using SPSS for Windows, version 23 (SPSS Inc., Chicago, IL, USA).

## Results

Of 105 patients initially included, four were excluded due to AF and two because of poor-quality TTE images. The final sample consisted of 99 patients: 21 with pIAB, 22 with aIAB, and 56 were patients with no IAB. ECGs were analyzed by one observer with low intra-observer variability (mean 0.6%; 95% CI 0.3–0.9%). The STE was analyzed in 95% of the acquired segments. Bland-Altman analysis showed good intra- and inter-observer agreement with a non-significant bias. Intra-observer variabilities were mean change 1.20% (95% CI, 1.10–1.31%) for LA global longitudinal strain and 1.36% (95% CI, 1.12–1.51%) for LA global longitudinal SR, and inter-observer variabilities were 1.32% (95% CI, 1.15–1.62%) and 1.6% (95% CI, 1.25–1.92%), respectively. Clinical and TTE characteristics of the patients included in this study are shown in Table 1 and Additional file 1: Table S1. Patients with aIAB were older and had a higher prevalence of diabetes. On TTE, patients with aIAB and pIAB had larger LAVImax compared to the patients with no IAB; Doppler echocardiography showed that the ASCTMF decreased and the E wave to A wave (E/A) ratio increased in the group of patients with pIAB and even more in the group with aIAB, compared to the patients with no IAB group. The values of strain and SR are shown in Table 2, with significant differences between the groups. We observed that the greater the degree of IAB, the lower the absolute values of LA wall deformation during the CT and R period of LA.

**Table 1:** Clinical and Doppler-echocardiographic characteristics

Variable	No ^a^IAB (c) (*n* = 56)	^b^pIAB (p) (*n* = 21)	^c^aIAB (a) (*n* = 22)	*p* ANOVA	*p* Post Hoc
Age, years	72.2 ± 8.0	69.7 ± 9.9	77.0 ± 7.6	0.014	*p* vs. *a* = 0.013
Men, n (%)	32 (57)	14 (67)	11 (50)	0.54	
Duration of P wave on ECG (ms)	108 (100–115)	127.5 (123–135.3)	139 (132–149.3)	< 0.001	c vs. *p* < 0.001
					c vs. *a* < 0.001
					*p* vs. *a* < 0.001
Hypertension, n (%)	37 (66)	17 (81)	19 (86)	0.09	
Diabetes mellitus, n (%)	16 (29)	9 (43)	14 (64)	0.011	
^d^LAVImax	25.6 (21.4–30.3)	33.5 (27.2–45.1)	35.2 (27.1–42.6)	< 0.001	c vs. *p* < 0.001
					c vs. *a* < 0.001
^e^E/A ratio	0.74 (0.66–0.87)	0.96 (0.79–1.3)	1.1 (0.85–1.3)	< 0.001	c vs. *p* = 0.011
					c vs. *a* < 0.001
^f^ASCTMF (%)	47 ± 37	44 ± 10	39 ± 10	0.006	c vs. *a* = 0.004

Clinical and Doppler-echocardiographic characteristics of the sample. Values are presented as mean ± SD or median (interquartile range). ^a^*IAB* interatrial block, ^b^*pIAB* partial interatrial block, ^c^*aIAB* advanced interatrial block, ^d^*LAVImax* Maximal left atrial volume indexed to body surface area (mL/m^2^), ^e^*E/A ratio* E wave to A wave ratio, a measure of the function of the left ventricle, ^f^*ASCTMF* atrial systolic contribution to total mitral flow

**Table 2:** Atrial strain and strain rate characteristics

Variable	No ^a^IAB (c) (n = 56)	^b^pIAB (p) (n = 21)	^c^aIAB (a) (n = 22)	*p* ANOVA	*p* Post Hoc
^d^ε peak ^e^LAc, (%)	− 2.8 (− 3.4 – − 2.1)	−2.9 (− 4.1 – − 1.9)	−1.9 (− 2.5 – − 0.68)	0.003	c vs. *a* < 0.001
					p vs. *a* = 0.011
^f^εs LAmax, (%)	18.0 ± 5.5	15.1 ± 3.0	12.7 ± 4.0	< 0.001	c vs. *p* = 0.020
					c vs. *a* < 0.001
^g^SRa, S^− 1^	−2.12 ± 0.57	− 1.20 ± 0.39	− 0.82 ± 0.26	< 0.001	c vs. *p* < 0.001
					c vs. *a* = 0.027
					p vs. *a* = 0.004
^h^SRs, S^− 1^	1.71 ± 0.27	0.99 ± 0.34	0.62 ± 0.25	< 0.001	c vs. *p* = 0.003
					c vs. *a* < 0.001
^i^SREF, S^− 1^	− 0.99 ± 0.57	− 0.94 ± 0.48	− 0.58 ± 0.22	0.009	c vs. p 0.003
					c vs. *a* < 0.001

Atrial strain and strain rate echocardiographic characteristics of the sample. Values are presented as mean ± SD or median (interquartile range). ^a^*IAB* interatrial block, ^b^*pIAB* partial interatrial block, ^c^*aIAB* advanced interatrial block, ^d^*ε* strain, ^e^*LAc* left atrial contraction, ^f^*εs LAmax* strain in LA reservoir phase, ^g^*SRa* strain rate in LA booster pump function phase, ^h^*SRs* strain rate in LA reservoir phase, ^i^*SREF* strain rate LA in early ventricular filling phase

Table 3 shows the standardized regression coefficients of the unadjusted simple linear regression model and the adjusted multiple linear regression model for potential confounders’ variables. The variables that showed the strongest associations with P-wave duration were LAVImax (direct association) and ASCTMF, εs LAmax, absolute SRa, and SRs (inverse associations). With these variables, we performed a backward stepwise analysis adjusting for age, gender, arterial hypertension, diabetes mellitus, LAVImax, and ASCTMF. The sample size allowed supporting all covariates from the linear model. In the first model, the SRs was associated with P-wave duration (standardized β − 0.345, *p* = 0.004). In the second model, LAVImax (standardized β 0.269, *p* = 0.013) and SRa (standardized β 0.420, *p* <  0.001) remained associated. In the third model, LAVImax (standardized β 0.319, *p* = 0.004) and εs LAmax (standardized β − 0.290, *p* = 0.008) remained associated.

**Table 3:** Unadjusted and adjusted standardized linear regression coefficients

	Unadjusted simple regression		Adjusted multiple regression^a^	
Variable	b Coefficient Standardized	*P*	b Coefficient Standardized	*P*
Age	0.220	0.029		
Gender	0.003	0.97		
Hypertension	0.181	0.07		
Diabetes mellitus	0.227	0.03		
^b^LAVImax (mL/m^2^)	0.353	< 0.001	0.347	< 0.001
^c^ASCTMF	−0.276	0.012	−0.227	0.039
^d^ε onset ^e^LAc, (%)	−0.347	< 0.001	− 0.184	0.07
ε peak LAc, (%)	0.152	0.14	0.088	0.40
^f^εs LAmax, %	−0.423	< 0.001	− 0.263	0.009
^g^SRa, S^−1^	0.520	< 0.001	0.369	< 0.001
^h^SRs, S^− 1^	−0.437	< 0.001	−0.296	< 0.001
^i^SREF, S^− 1^	0.249	0.018	0.161	0.12

Unadjusted and adjusted multiple standardized linear regression coefficients^a^: adjustment for variables age, gender, hypertension, diabetes mellitus, LAVImax, and ASCTMF. ^b^*LAVImax* Maximal left atrial volume indexed to body surface area (mL/m^2^), ^c^*ASCTMF* atrial systolic contribution to total mitral flow, ^d^*ε* strain, ^e^*LAc* left atrial contraction, ^f^*εs LAmax* strain in LA reservoir phase, ^g^*SRa* strain rate in LA booster pump function phase, ^h^*SRs* strain rate in LA reservoir phase, ^i^*SREF* strain rate LA in early ventricular filling phase

**Fig. 3:**
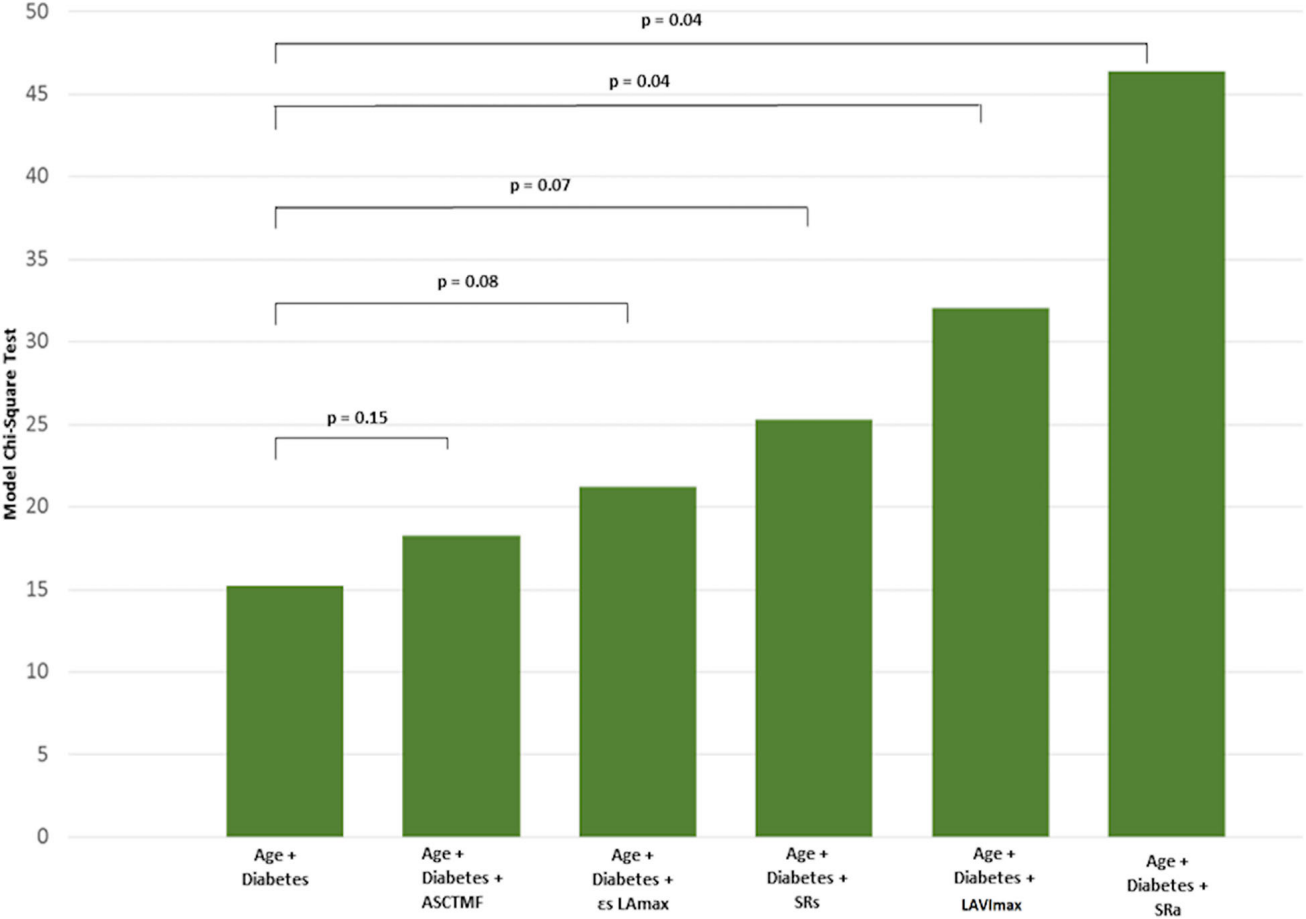
Comparison of multivariate models. Incremental correlation between interatrial block and echocardiographic variables with respect to age and diabetes mellitus. We used the multinomial logistic regression model, with the dependent variable taking three values (“pIAB,” “aIAB,” and “patients with no IAB”). pIAB = partial Interatrial Block; aIAB = advanced Interatrial Block; ASCTMF = atrial systolic contribution to total mitral flow; LA = left atrial; εs LAmax = LA strain in reservoir phase; SRs = LA strain rate in reservoir phase; LAVImax = maximal LA volume indexed to body surface area; SRa = LA strain rate in booster pump function phase

**Fig. 4:**
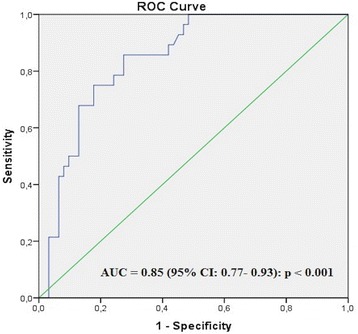
Receiver operating characteristic curves of the atrial booster pump function phase (SRa) (independent variable) in correlation with presence or not of interatrial block (dependent variable). AUC = area under the curve

Table 4 shows the variables significantly associated with both types of IAB after univariate multinomial regression analysis. Several multinomial logistic sub-models were constructed to avoid saturation and collinearity (Additional file 2: Table S2) of the regression model. The reference model comprised age and diabetes mellitus, which were included in successive models with the following variables introduced one by one: LAVImax, ASCTMF, εs LAmax, SRa, and SRs (Table 5). Comparing the overall differences between five nested models, the models which included LAVImax and SRa proved statistically superior to the others (Fig. 3). The results of the linear and logistic models are similar, suggesting in a robust way the association between P-wave of ECG duration or type of IAB and the mean peak of SRa and SRs. According to the ROC curve analysis (Fig. 4), the best cut-off that identified patients with IAB versus patients with no IAB was absolute SRa ≤ − 1.20 S^− 1^ (AUC 0.85, CI 0.77–0.93, *p* <  0.001), with a sensitivity of 70% (CI: 54.6–81.9) and specificity of 78% (CI: 64.8–87.2). On grouping the total sample of SRa by tertiles and observing the distribution, according to the presence or absence of IAB, a linear trend was demonstrated, such that the lower the absolute value of SRa, the greater the number of patients with IAB (Mantel Haenszel Test for tendencies *p* <  0.001) (Additional file 3: Table S3).

**Table 4:** Multinomial univariate analysis results

	^a^pIAB			^b^aIAB		
Variable^c^	^d^OR	95% ^e^CI	*P*	OR	95% CI	*P*
Age	0.96	0.91–1.02	0.25	1.1	1.021–1.15	0.026
Gender	0.67	0.23–1.91	0.45	1.3	0.50–3.60	0.57
Hypertension	2.10	0.61–7.04	0.25	4.62	0.97–22.04	0.06
Diabetes mellitus	1.83	0.64–5.18	0.26	4.87	1.66–14.32	0.004
^f^LAVImax(mL/m^2^)	1.11	1.05–1.17	< 0.001	1.08	1.03–1.14	0.004
^g^ASCTMF	0.018	0.01–12.7	0.04	0.01	0.001–0.03	0.02
^h^εs LAmax, %	0.90	0.78–0.99	0.04	0.76	0.65–0.88	< 0.001
^i^SRa, S^−1^	8.03	2.16–29.9	< 0.001	16.5	5.94–46.2	< 0.001
^j^SRs, S^−1^	0.04	0.04–0.31	< 0.001	0.009	0.001–0.1	< 0.001

^a^*pIAB* partial interatrial block, ^b^*aIAB* advanced interatrial block, ^c^*Variable* normal ECG group taken as reference category. ^d^*OR* odds ratio, ^e^*CI* confidence interval, ^f^*LAVImax* Maximal left atrial volume indexed to body surface area (mL/m^2^), ^g^*ASCTMF* atrial systolic contribution to total mitral flow, ^h^*εs LAmax* strain in LA reservoir phase, ^i^*SRa* strain rate in LA booster pump function phase, ^j^*SRs* strain rate in LA reservoir phase

**Table 5:** Multinomial multivariate analysis results

	^a^pIAB			^b^aIAB		
Variable^c^	^d^OR	95% ^e^CI	*P*	OR	95% CI	*P*
^f^LAVImax (mL/m^2^)	1.12	1.05–1.18	< 0.001	1.10	1.04–1.17	0.002
^g^ASCTMF	0.02	0.003–11.1	0.24	0.03	0.004–0.14	0.003
^h^εs LAmax	0.85	0.72–1.01	0.07	0.77	0.64–0.92	0.004
^i^SRa	11.5	2.7–49	0.001	98.2	16–120.4	< 0.001
^j^SRs	0.03	0.003–0.29	0.003	0.008	0.001–0.12	0.004

^a^*pIAB* partial interatrial block, ^b^*aIAB* advanced interatrial block, ^c^*Variable* normal ECG group taken as reference category. ^d^*OR* odds ratio, ^e^*CI* confidence interval, ^f^*LAVImax* Maximal left atrial volume indexed to body surface area (mL/m^2^), ^g^*ASCTMF* atrial systolic contribution to total mitral flow, ^h^*εs LAmax* strain in LA reservoir phase, ^i^*SRa* strain rate in LA booster pump function phase, ^j^*SRs* strain rate in LA reservoir phase

## Discussion

Our main finding was that a longer duration of the P-wave in the ECG, belonging to the group of patients with pIAB and aIAB, correlated directly with the increase in LAVImax on 2D echocardiogram (2DE) and inversely with the decrease in the absolute value of LA SRa and SRs on STE (Fig. 1d). To our knowledge, our study is the first to describe LA wall deformation alterations determined using STE in patients with IAB. The SRa was especially useful for correlation with the presence or absence of IAB. This indicates the reliable correlation between ECG and STE in patients with IAB.

Several studies using TTE have shown increased LA size and slow and poor contractility in patients with IAB [3, 4, 12]. Similar findings were observed in our patients with IAB, in relation to the LA volume increase, especially in those with aIAB, where ASCTMF was significantly decreased compared to in patients with no IAB. Although IAB and enlarged LA share a similar ECG pattern, a recent study has indicated that they are independent entities [3].

The European Association of Cardiovascular Imaging/European Heart Rhythm Association Expert Consensus for the evaluation of patients with AF indicates that, in this context, measuring LA strain using STE is very promising [21], even in patients with permanent AF [16]. Sun et al. analyzed the effect of aging on the strain of the LA using 2DE STE, noting that, as age increases, the absolute value of SRa increases, regardless of gender. This is due to coupling between LA CT and decreased diastolic function with older age [20]. In our series, patients with aIAB were older than the subjects in the other two groups, despite the fact that their absolute SRa values were pathologically diminished (Tables 1 and 2), probably due to age-related atrial fibrosis [22]. However, not only age must be considered, because the regression analyses adjusted for this and the other confounding variables showed correlations with both types of IAB. In addition, absolute values of SRa showed a linear decrease in the groups of patients with pIAB and aIAB.

Speckle tracking echocardiography findings of LA deformation similar to ours have been reported in patients with permanent or PAF [16]. Hirose T et al. reported that subjects who develop new-onset non-valvular AF have lower absolute values of SRa and SRs than those who do not [11]. Other authors have evaluated the role of LA deformation imaging as a predictor of a successful second cardiac ablation in PAF, noting that strain and SR during the CT and R periods of the LA were significantly lower in the second cardiac ablation group compared to healthy volunteers and patients undergoing first cardiac ablation without AF recurrence (*p* <  0.001, for all) [23]. It has also been shown that a decrease in the LA global longitudinal strain is an independent predictor of the progression of PAF towards persistent or permanent AF [24].

Atrial fibrosis can be visualized and quantified using cardiac magnetic resonance imaging (CMR) with late gadolinium enhancement (LGE), but it can also manifest as atrial enlargement, decrease in deformation during CT and R periods, determined using LA 2DE STE, even before the occurrence of atrial dilation, as well as higher daily AF load [25]. All these findings have been associated with stroke in patients with AF [26]. One possible application of determining atrial strain could be to predict the risk of AF. This has recently been demonstrated by Mahnkopf et al., who assessed LA fibrosis with LGE-CMR and atrial function using STE before cardiac ablation, creating the Utah classification. They correlated AF recurrence directly with decreased εs LAmax and the highest degree of atrial fibrosis on LGE-CMR [27]. Therefore, the STE 2DE finding of decreased LA deformation in our patients with IAB is identical to that predicting AF in patients with no IAB [2, 22]. Given the direct relationship between decreased deformation and LA fibrosis in PAF, this same relationship could exist between decreased deformation and LA fibrosis in our patients with IAB. The latter, associated with the LA enlargement in the patients with IAB, would indicate that these patients present the two factors that define atrial structural remodeling [28]. The Expert Consensus on Atrial Cardiomyopathy proposed the following as a working definition of this: “any complex of structural, architectural, contractile, or electrophysiological changes affecting the atria with the potential to produce clinically-relevant manifestations.” It could, therefore, be considered that the contractile alterations of the LA wall and the occurrence of supraventricular arrhythmias in patients with IAB would be a form of atrial cardiomyopathy [28]. The atrial myopathy hypothesis proposes that atrial fibrosis increases the risk of thromboembolism independently of atrial rhythm [29]. Identifying these STE parameters in patients with IAB is important because they indicate alteration of atrial structure and function, even before severe enlargement of the LA occurs, as we have detected in pIAB. It remains to be clarified whether this decrease, as determined using STE in IAB, has a similar ability to predict the development of new-onset AF.

### Limitations

We have not been able to establish whether IAB is a degenerative process independent of the specific atrial conduction system or associated with atrial fibrosis. We did not measure the LA electromechanical coupling, the LA mechanical dispersion, or the inter- and intra-atrial electromechanical delays. Failure to perform LGE-CMR prevented us from directly correlating the degree of fibrosis with the 2DE STE findings of LA contractile function. The three-dimensional TTE technique could provide useful strain data from multiple sites and a more accurate assessment of atrial volumes. Finally, and because our study was not designed to establish a causal relationship between decreased atrial deformity and AF, a prospective longitudinal design would be needed to determine whether ECG and STE findings in patients with IAB are related to AF and cardioembolic events. Then, establishing the incremental value that STE has on the ECG can provide information regarding prognosis and therapeutic measures in patients with IAB.

## Conclusions

To our knowledge, this is the first study to describe LA wall deformation alterations determined using 2D STE in patients with IAB. It demonstrated that SRa and SRs of LA are pathologically diminished in patients with IAB. This finding could indicate the existence of an atrial cardiomyopathy in patients with IAB.

## Additional files

Additional file 1: **Table S1.** Clinical and Doppler-echocardiographic characteristics. Clinical and transthoracic echocardiography characteristics of the patients included in this study. (DOC 47 kb)

Additional file 2: **Table S2.** Matrix of correlations between P-wave duration on ECG, Doppler- echocardiographic and strain variables. Correlation between continuous variables: P-wave duration on ECG, Doppler- echocardiographic and strain variables. (DOC 41 kb)

Additional file 3: **Table S3.** Peak left atrial longitudinal strain rate in booster pump function phase in tertiles. Mantel Haenszel Test for tendencies of peak left atrial longitudinal strain rate in booster pump function phase. (DOC 34 kb)

## Abbreviations

AF: Atrial fibrillation
ASCTMF: Percentage of atrial systolic contribution to total mitral flow
BSA: Body surface area
CD: LA conduit period;
CIs: 95% confidence intervals
CMR: Cardiac magnetic resonance imaging
CT: LA contraction period
D: Diastasis
EF: LV early filling
ER: Early reservoir period
IAB: Interatrial block
LA: Left atrium
LAVImax: Maximal LA volume indexed to body surface area
LGE: Late gadolinium enhancement
LV: Left ventricular
MVC: Mitral valve closure
MVO: Mitral valve opening
OR: Odds ratio
PAF: Paroxysmal atrial fibrillation
R: LA reservoir period
ROC: Receiver operating characteristic curves
SR: Strain rate
SRa: Peak left atrial longitudinal strain rate in booster pump function phase
SRs: Peak left atrial longitudinal strain rate in early reservoir phase
STE: Speckle tracking echocardiography
TTE: Transthoracic echocardiography
εs LAmax: Maximal peak left atrial longitudinal strain in reservoir phase
