# Supplementary material for: Interatrial block and atrial remodeling assessed using speckle tracking echocardiography
Source: BMC Cardiovasc Disord. 2018 Feb 21;18:38. doi: 10.1186/s12872-018-0776-6 (PMC5822665; doi:10.1186/s12872-018-0776-6)
Supplement: Supplementary file 1 — Table S1. Clinical and Doppler-echocardiographic characteristics. Clinical and transthoracic echocardiography characteristics of the patients included in this study. (DOC 47 kb) [file 12872_2018_776_MOESM1_ESM.doc]

**Additional file**

**Table S1. Clinical and Doppler-echocardiographic** characteristics.

| **Variable** | **^*^IAB No (c)**  **(n = 56)** | **✝pIAB (p)**  **(n = 21)** | **ǂaIAB (a)**  **(n = 22)** | **p**  **ANOVA** | **p**  **Post Hoc** |
| --- | --- | --- | --- | --- | --- |
| §ARBs or ^ǁ^ACEIs, n (%) | 10 (18) | 6 (29) | 5 (23) | 0.57 |  |
| Beta-blockers, n (%) | 20 (36) | 11 (52) | 10 (45) | 0.12 |  |
| Heart failure, n (%) | 3 (5) | 4 (19) | 1 (4) | 0.12 |  |
| Valve disease, n (%) | 13 (23) | 7 (33) | 7 (32) | 0.54 |  |
| Coronary disease, n (%) | 11 (20) | 6 (29) | 9 (41) | 0.12 |  |
| ^#^LV^**^EDV/^††^BSA, (mL/m^2^) | 47.3 ± 16.1 | 59.6 ± 20.0 | 46.1 ± 9.6 | 0.017 | c vs p 0.017 |
| LV^‡‡^ESV/BSA, (mL/m^2^) | 13.9 ± 7.1 | 20.7 ± 13.8 | 12.3 ± 4.2 | 0.004 | p vs a 0.045 |
| ^§§^LVEF (%) | 71.1 ± 9.1 | 69.0 ± 11.5 | 74.4 ± 4.8 | 0.15 |  |
| E velocity, (cm/s) | 75.4 ± 20 | 81.5 ± 20.4 | 89.8 ± 21.2 | 0.02 | c vs a 0.017 |
| A velocity, (cm/s) | 92.3(80.4-119.1) | 70.1 (61-90.4) | 77.3 (63-102.6) | 0.09 |  |
| E/e´ | 13 (11.1-16.1) | 11.9 (8.3-14.3) | 15 (11.1-17.4) | 0.06 |  |
| LV mass index, (g/m^2^) | 104 ± 37.6 | 109 ± 39.0 | 118 ± 45.5 | 0.40 |  |
| ^ǁǁ^BMI, (kg/m^2^) | 27.5 ± 3.7 | 28.2 ± 4.4 | 27.0 ± 4.4 | 0.67 |  |

Values are mean ± SD or median (interquartile range).

^*^IAB = interatrial block; ✝pIAB = partial interatrial block; ǂaIAB = advanced interatrial block; ^§^ARBs = angiotensin receptor blockers; ^ǁ^ACEIs = angiotensin converting enzyme inhibitors; ^#^LV = left ventricle; ^**^EDV = end diastolic volume; ^††^BSA = body surface area; ^‡‡^ESV = end systolic volume; ^§§^LVEF = left ventricular ejection fraction; ^ǁǁ^BMI = body mass index.
