# Supplementary material for: Interatrial block and atrial remodeling assessed using speckle tracking echocardiography
Source: BMC Cardiovasc Disord. 2018 Feb 21;18:38. doi: 10.1186/s12872-018-0776-6 (PMC5822665; doi:10.1186/s12872-018-0776-6)
Supplement: Supplementary file 2 — Table S2. Matrix of correlations between P-wave duration on ECG, Doppler- echocardiographic and strain variables. Correlation between continuous variables: P-wave duration on ECG, Doppler- echocardiographic and strain variables. (DOC 41 kb) [file 12872_2018_776_MOESM2_ESM.doc]

**Additional file 2**

**Table S2. Matrix of correlations between P-wave duration on ECG, Doppler- echocardiographic and strain variables.**

| **Variable** | ^*^**LAVImax** | ^✝^**ε onset LAc** | **ε peak** ^ǂ^**LAc** | ^§^**εs LAmax** | ^ǁ^**SRa** | ^#^**SRs** |
| --- | --- | --- | --- | --- | --- | --- |
| **P-wave duration on ECG**  **Correlation coefficient**  **Bilateral significance** | 0.39^*^  < 0.001 | -0.33^*^  0.001 | 0.21^*^  0.04 | -0.41^*^  < 0.001 | 0.60^*^  < 0.001 | -0.51^*^  < 0.001 |
| **E/A ratio**  **Correlation coefficient**  **Bilateral significance** | 0.29^*^  0.008 | -0.32^*^  0.004 | 0.19  0.08 | -0.26^*^  0.02 | 0.55^*^  < 0.001 | -0.38^*^  0.001 |
| **E/e´**  **Correlation coefficient**  **Bilateral significance** | 0.1  0.38 | -0.19  0.09 | 0.17  0.13 | -0.21  0.06 | 0.30^*^  0.008 | -0.22  0.07 |
| ^✝✝^**ASCTMF**  **Correlation coefficient**  **Bilateral significance** | -0.15  0.19 | 0.21  0.06 | -0.27^*^  0.02 | 0.42^*^  < 0.001 | -0.54^*^  < 0.001 | 0.44^*^  < 0.001 |
| ^*^**LAVImax**  **Correlation coefficient**  **Bilateral significance** |  | -0.25^*^  0.03 | -0.08  0.48 | -0.25^*^  0.02 | 0.29^*^  0.008 | -0.25^*^  0.03 |

^*^LAVImax = Maximal left atrial volume indexed to body surface area (mL/m^2^); ^✝^ε = strain; ^ǂ^LAc = left atrial contraction; ^§^εs LAmax = maximal left atrial longitudinal strain in reservoir phase; SR = strain rate; ^ǁ^SRa = peak left atrial longitudinal strain rate in booster pump function phase: ^#^SRs = peak left atrial longitudinal strain rate in early reservoir phase; ^✝✝^ASCTMF = atrial systolic contribution to total mitral flow.

*Asterisk indicates significant correlation between variables.
