# Supplementary material for: Interatrial block and atrial remodeling assessed using speckle tracking echocardiography
Source: BMC Cardiovasc Disord. 2018 Feb 21;18:38. doi: 10.1186/s12872-018-0776-6 (PMC5822665; doi:10.1186/s12872-018-0776-6)
Supplement: Supplementary file 3 — Table S3. Peak left atrial longitudinal strain rate in booster pump function phase in tertiles. Mantel Haenszel Test for tendencies of peak left atrial longitudinal strain rate in booster pump function phase. (DOC 34 kb) [file 12872_2018_776_MOESM3_ESM.doc]

**Additional file 3**

**Table S3. Peak left atrial longitudinal strain rate in booster pump function phase in tertiles.**

| **Level of Exposure** | **≤ - 1.70** | **- 1.69 - - 1.0** | **< - 0.99** | **Total** |
| --- | --- | --- | --- | --- |
| **^*^IAB** | 2 | 14 | 24 | 40 |
| **No IAB** | 28 | 17 | 5 | 50 |
| **Total** | 30 | 31 | 29 | 90 |
| **Pseudo-ODDs** | 0.071 | 0.824 | 4.8 | 0.8 |
| **Last level** ^✝^**OR** | 0.015 | 0.172 | 1.0 |  |
| **95% ^ǂ^CI Lower** | 0.003 | 0.052 |  |  |
| **95% CI Upper** | 0.084 | 0.567 |  |  |

MH Test for Trend: Z = 5.8482 (p < 0.001)

^*^IAB = interatrial block; ^✝^OR: odds ratio; **^ǂ^**CI: confidence interval.
